# Using Novel Multi-Frequency Analysis Methods to Retrieve Material and Temperature Information in Tactile Sensing Areas

**DOI:** 10.3390/s22228876

**Published:** 2022-11-17

**Authors:** Mehdi Abdelwahed, Lounis Zerioul, Alexandre Pitti, Olivier Romain

**Affiliations:** 1 ETIS, CY Cergy Paris University, ENSEA, CNRS UMR 8051, 95000 Cergy, France; 2 Institut VEDECOM, 78000 Versailles, France

**Keywords:** tactile sensor, artificial skin, electrical impedance tomography, multi-frequencies, machine learning, material recognition, temperature

## Abstract

This article presents a novel artificial skin technology based on the Electric Impedance Tomography (EIT) that employs multi-frequency currents for detecting the material and the temperature of objects in contact with piezoresistive sheets. To date, few artificial skins in the literature are capable of detecting an object’s material, e.g., wood, skin, leather, or plastic. EIT-based artificial skins have been employed mostly to detect the position of the contact but not its characteristics. Thanks to multi-frequency currents, our EIT-based artificial skin is capable of characterising the spectral profile of objects in contact and identifying an object’s material at ambient temperature. Moreover, our model is capable of detecting several levels of temperature (from −10 up to 60 °C) and can also maintain a certain accuracy for material identification. In addition to the known capabilities of EIT-based artificial skins concerning detecting pressure and location of objects, as well as being low cost, these two novel modalities demonstrate the potential of EIT-based artificial skins to achieve global tactile sensing.

## 1. Introduction

Several techniques have been used to endow artificial skins with multiple tactile modalities, including contact pressure, texture discrimination and temperature detection [1]. One challenge of most artificial skin designs is to combine as many different sensors into the same device [2]. However, difficulties arise when it comes to making large surfaces, increasing the complexity of the electronic architecture. The opposite approach, more minimalist, is to employ only one medium [3], e.g., a piezoresistive sheet of composite material, to retrieve pressure localisation information. The advantages of using this technique include easier conception, low cost and less energy consumption [4,5]. Here, difficulties arise concerning retrieving the localisation of the touch, i.e., inferring the desired information from the medium during reconstruction. Multiple phenomena occur as they are all intertwined in the signal (pressure, shape, location, temperature, material, texture). One popular technique applying this approach is Electrical Impedance Tomography (EIT), which uses an electrical signal to carry out pressure information from a large surface medium [6]. In tactile devices, a voltage matrix is retrieved when a current is injected in a piezoresistive layer. By retrieving the conductivity changes, the localisation of the contact is inferred by the local change of conductivity. Despite its advantages in terms of energy consumption and practical use, an EIT-based technique has poor spatial precision and can be influenced by the material conductivity in contact [7].

**Figure 1:**
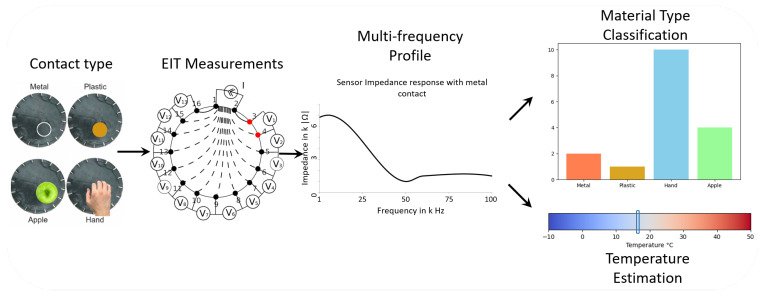
Diagram of the sensor global architecture: from the initial contact, identification allows retrieval of material type and temperature. Measurements are done with multiple frequencies and four types of objects at different temperatures. The aim is to identify the object type and estimate the contact temperature.

The EIT reconstruction is an ill-posed parametric problem due to the finite number of electrodes used and the nature of the inverse problem of the conductivity reconstruction [8]. This also involves a long computational method to retrieve the reconstruction, thus making it unsuitable for real-time applications. However, this can be solved by using machine learning techniques [9,10,11,12,13,14]. To tackle some of the issues, we extended the traditional EIT-based tactile sensor to use Multi-Frequency Analysis to retrieve more information. This can be compared to how the human somatosensory system processes tactile information [15,16]. The touch sense is recomposed from the Multi-Frequency Analysis made from several mechanoreceptors, ranging from a few Hertz (5 to 20 Hz) to a few thousand (1000 to 2000 Hz) [17,18]. These different frequencies are used to distinguish between different tactile information. Moreover, the frequencies also allow encoding information while it is being transmitted to the brain. Because of that, the touch sense is capable of retrieving different information types, from caresses to high pressure with pain and temperature as well. Inspired by this biological mechanism, we propose to extend the standard EIT method for artificial skin using a frequency spectrum to reconstruct different modalities such as pressure, temperature and material type. In the context of artificial skins, by comparison, the EIT technique usually employs a direct current with no frequency to reconstruct localisation and pressure. We develop an artificial skin capable of retrieving localisation, pressure and material type, with the ability to be scaled to cover large surfaces. Figure 1 shows the global architecture of our sensor. With our machine learning approach, we extend this ability by retrieving temperature information. During our experiments, we showed the pertinence of our approach with the detection of multiple materials with different conductivity profiles in the multi-frequency spectrum, as well as their temperature. The experiments also showed accurate discrimination from low conductivity materials (plastic, biological tissues) to high conductivity materials (metal). Our approach resolves the drawback of the conventional EIT technique for artificial skins for not detecting different contact types easily at the surface and extends it with unprecedented features: material and temperature detection. Multispectral EIT gives the promise of designing multi-modal artificial skins using one sensor only.

The paper is organised as follows; In Section 2, we present state of the art impedance spectroscopy techniques using multi-frequencies, the EIT technique and our approach using multi-frequency EIT. In Section 3, we present the device, the hardware architecture and the EIT injection pattern used. The reconstruction algorithm is also presented. In Section 4, we present the three experiments using multi-frequency EIT to extract feature characteristics of the material, the pressure and the temperature using plastic, metal and human skin. Finally, in Section 5, we discuss the potential uses of this sensor and its advantages in future human–machine Interactions toward the design of a multi-modal tactile sensor.

## 2. Methods

This section will present current flexible tactile sensors and the EIT technique with its applications. EIT is based on the impedance of the material in order to reconstruct the conductivity. It is an ill-posed problem; therefore, most of the recent research is focused on the reconstruction aspect.

### 2.1. Flexible Sensors

From medicine to robotics, research about flexible sensors has flourished [19,20]. The flexibility aspect is challenging; however, many different materials are available [21]. They can be related to different technologies based on electrical phenomena occurring: piezoresistive, piezoelectric, piezocapacitive and triboelectric [20,22,23]. For example, PDMS (polydimethylsiloxane) is one of the materials used to design flexible sensors. Each has different abilities and produces different behaviours. They can be very low cost, have good stretch capabilities and can give localisation and pressure information.

Table 1 summarises different sensor technologies. Our sensor is based on a carbon black polymer fabric called Velostat/Linqstat from 3 M. It has a surface resistivity of 31 kΩ/sq·cm. The working temperature range is from −45 to 65 °C. It is low-cost and widely available. This is very advantageous compared to developing custom fabric with specific characteristics. However, it suffers from a lack of stability with high hysteresis and bad ageing and cannot stand very high temperatures (80 °C) or it faces the risk of irremediable electrical property changes.

The influence of the temperature on the resistivity is not known for the Velostat. However, because it is a carbon-black-based polymer, the sensor behaviour can be deduced from it. The carbon-black material impedance is directly correlated with the temperature [30]. This research shows the theoretical and experimental results for temperature and density influences of carbon-black impedance. In our case, because Velostat is a commercial product, the exact composition is not known. Conjectures can be made, but we decided to conduct our own experiments to measure the temperature influence.

### 2.2. Electrical Impedance Tomography

The EIT is an imagery technique born in the mid-1980s [31,32,33]. The idea is to retrieve information about a hidden object. For example, the position of a certain object within another is retrieved. The way to retrieve this information is to inject an electrical current (or tension) in a certain area of the object and measure the tension (or the current) in another area. This gives information about the conductivity of the area. If this step is repeated across the object, a conductivity map can be reconstructed. If Ω is a bounded area (of dimensions d≥2) and ω is its frequency, then the problem can be described as follows:

(1) γ(x,ω)=σ(x)+iωϵ(x),

where σ is the electrical conductivity and ϵ is the electrical permittivity at position *x*. γ is the inverse electrical impedance at position *x* of Ω; thus, the EIT is the inverse problem of obtaining the impedance of Ω with only current or voltage measurement from the boundaries of Ω.

EIT systems are based on the Electronic Impedance Spectroscopy (EIS) technique. A typical EIS system consists of an electronic device that generates stimulations over different frequencies and measures the electrical signal coming from a Sample Under Test (SUT). Usually, metallic electrodes that have physical contact with the SUT are used. The most common implementation, even in commercial instruments, is the frequency sweep technique. Here, the stimulation signal is a sine wave where the frequency is modified in each impedance calculation. There are several challenges for the integration of rapid measurement for tactile sensors by EIS. The frequency sweep technique requires an extensive exploration time, especially at lower frequencies. For the measurements, acquisition time should be shorter than the time between successive touches. Therefore, both faster and accurate methods are needed. Nowadays, EIT is used for different applications in the medical field, from tumour detection to brain stroke monitoring [34]. However, the first and most used application is lung monitoring in ICU. This is because of the advantage EIT has compared to others: non-invasiveness.

### 2.3. Multi-Frequency

For certain applications, one frequency is not enough. For detection of two material types, such as blood/bones in the body, multi-frequencies are necessary. In this case, a solution will be to apply two tomographies with different frequencies. A straightforward approach is to inject, frequency per frequency, all the frequencies needed for the reconstruction. This method can be very effective if the situation needs only a couple of frequencies but can be very slow when more frequencies are injected. The sweep frequency is an extension of this method, but the fastest way is to inject all frequencies at once. However, the complexity increases in this setup.

### 2.4. Current Injection in EIT

EIT is based on an injected current in a body to reconstruct its conductivity. However many methods can be used to obtain these results. The most basic one is to use a Direct Current (DC) signal. No frequency is used, so it is called Electrical Resistivity Tomography [35]. It is faster to obtain results from this approach but for some applications, the frequency plays a crucial role in the reconstruction: depending on the frequency, a reconstruction of a specific material can have a better resolution.

### 2.5. Reconstruction

The reconstruction algorithm is the step that produces the conductivity distribution of a body from EIT. This can be used to obtain the spatial localisation of the contact in our setup, for example. By using an approximation of Maxwell’s equations, we have:

(2) $$\begin{array}{cc} \; & ▽H(x,\omega )=\gamma (x,\omega )E(x,\omega ),\\ \; & ▽E(x,\omega )=0. \end{array}$$

*H* is the magnetic field strength and *E* the electric field. By setting the differential magnetic field strength as the electrical current and the electric field as the differential scalar electric potential, we obtain:

(3) $$\begin{array}{cc} \; & E(x,\omega )=-▽\phi (x,\omega ),\\ \; & ▽H(x,\omega )=j(x,\omega ), \end{array}$$

Using this, we can obtain from Equation (2) Ohm’s Law:

(4) j(x)=−γ(x,ω)▽ϕ(x,ω).

Finally, Equation (4) gives us the possibility of writing the partial differential equation:

(5) ▽[γ(x,ω)▽ϕ(x,ω)]=0.

The inverse conductivity problem is to find γ(x,ω) from Equation (5). In order to do that, several techniques have been developed. The common method applied is parametric reconstruction. This uses techniques such as the back-projection method or the iterative method. Most parametric methods have been integrated in EIDORS, a library found in Matlab that implements some basic and improved reconstruction methods [36]. This used the Complete Electrode Model (CEM), Finite Element Methods (FEM) and other parametric tools to reconstruct the conductivity and tackle the ill-posed problem. Methods such as GREIT [37] and PEPR [38] try to compensate for this problem.

Another type of reconstruction is to learn the reconstruction using probabilistic methods [39,40] and machine learning [41,42]. The advantage is that a complete description of the setup is not necessary, having enough data is the only requirement for this technique.

## 3. Sensor

### 3.1. The Tactile Sensor

**Figure 2:**
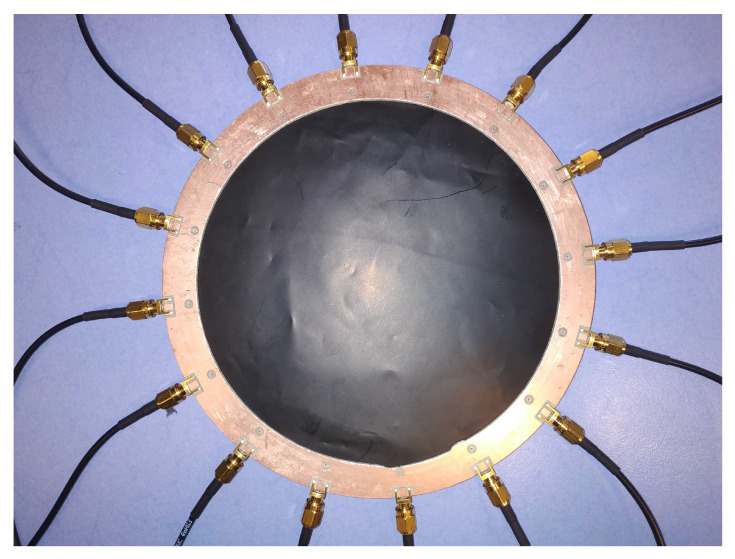
Top view of the tactile sensor. The black layer is the Velostat and electrodes are clamped to it but only on the top face of the Velostat. This assures the measurement is only related to the surface impedance changes.

The Velostat fabric itself is shaped in a circle of 20 cm in diameter, as seen in Figure 2. The electrodes are clipped to the boundaries of the fabric circle uniformly. To have only the surface resistivity playing a role, the Velostat fabric is put on a small layer of foam and a rigid non-conductive base. These three layers are clipped together by the electrodes themselves. The electrodes are then connected to the multiplexing card. Because the injection pattern uses two electrodes and the measurement also uses two electrodes, a total of four multiplexers is needed.

**Figure 3:**
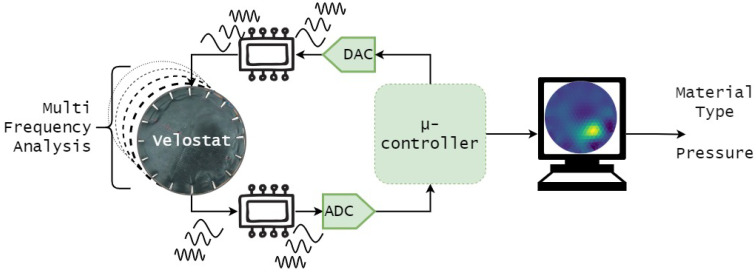
Diagram of the sensor global architecture, the three parts are the fabric called Velostat, the signal conditioning which is composed of the multiplexing part and the DAC/VCCS. The last part is handled with the microcontroller and the data are processed with a computer.

The sensor can be divided into three parts: The sensor itself, the signal conditioning and the data processing part. The first two parts work together to obtain the voltage acquisition of the sensor and the processing part allows obtaining the categorisation of the material type and other information. Figure 3 shows the three parts working together.

The sensing area is designed as a multiple layer scheme with the Velostat on top. Below the first layer, foam is placed to increase the bend when a force is applied to the Velostat and to add softness to the sensor. On the bottom part, an insulating layer is put before the bottom layer which composes the support layer. We use cardboard as the insulating layer and some copper/plastic for the support layer. The total thickness of the sensor is about 1 cm.

Our sensor has a diameter of 20 cm, which is an arbitrary choice. Tests have been made with different sizes, especially smaller sizes. Our purpose is to build a large-scale sensor and 20 cm seems to be large enough in our case. Larger areas can also be developed using this technology, but for simplicity and because the purpose is to highlight the multi-modality capabilities, tests were conducted with only one sensor size.

### 3.2. Electrical Hardware

The first part is composed of a piezoresistive film conducting electricity. The film is cut in a round shape and connects to 16 electrodes. The electrodes are connected to the signal conditioning part. This part is composed of a multiplexing/demultiplexing stage, a Voltage-Controlled Current Source (VCCS) and a microcontroller. The signal is first generated by the microcontroller as a sinusoidal (at 1 k, 2.5 k, 5 k, 7.5 k, 10 k, 25 k, 50 k, 75 k, 100 k and 250 kHz) with a Voltage Peak-to-Peak (VPP) to 2 V and a DC component of 1 V. The signal is then processed by the VCCS, converting it to a constant 100 μA. The current obtained is steered by the multiplexing stage to produce a current in the sensor between two electrodes. The microcontroller then commands the demultiplexing to obtain two electrodes as voltage measurement. This measurement is then passed to the third part. The data are processed afterward for categorisation.

### 3.3. Signal Processing

To inject a current into the sensor, it needs to be constant first. A DAC generates the waveform first. This comes from the microcontroller itself, an Analog Discovery 2. It can generate and measure analogue signals, control the multiplexing part with digital I/O pins and also communicate with a computer using a USB interface. The generated waveform is passed to a Howland Current Pump. The circuit was designed to obtain a VCCS with a constant current of 100 μA. This allows having a bipolar signal with a constant current. Because we use EIT with current injection, we need to provide a constant alternative current. This means the voltage is measured across the electrodes using the multiplexing component.

### 3.4. Data Processing

This part allows the retrieval of the data from the signal processing part to the microcontroller. The data are then saved in a computer for offline processing. Real-time processing by the computer is possible but was not implemented in this study. The retrieved data were sampled at 10 times the frequency of the stimuli. A complete frame, which means all the frequency with all the measurements is carried out in under 30 s for a standard, frequency by frequency, measurement. Another mode using frequency sweep retrieves data in under 5 s. The data are finally processed to categorise information about material types, pressure and temperature.

### 3.5. Learning Technique

**Figure 4:**
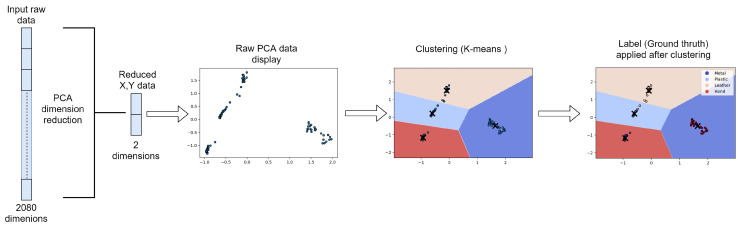
Diagram of the sensor identification architecture, The data were reduced with a PCA. Then the reduced data were categorise using non supervised learning (K-means). The data were then labelled and are shown.

In this research, the machine learning approach was used to process the data. However, unlike previous works [41], the learning approach was used to discriminate material pressure and also describe temperature drift. This technique was also used in complementary of multi-frequency injection (see Figure 4). First, the method consisted in lowering the data dimension. This was accomplished with a Principal Component Analysis (PCA). Then the data were given in a K-Nearest Neighbour classification. Other methods were used in this step such as Support Vector Machine (SVM) and Gaussian Mixture Model (GMM). The purpose was to classify the stimuli by their type, their pressure or their temperature. With this technique, prior learning can be used to classify afterward, but new classes can also emerge and be learned with this technique. Some drawbacks are prior learning must be robust enough to work with new data and it does not allow reconstructing the contact spatially. The data after PCA were plotted alongside the centroids of the k-means and the decision boundaries. The data were labelled afterward in order to visually represent the categorisation.

## 4. Experiments

In this section, three experiments will be presented. The first one describes the sensor behaviour with different contact pressure. The second one will highlight the differences in results based on changes in temperature. Finally, the last experiment will show material recognition with pressure and temperature changes.

Previous experiments show [9] the ability to recognise material types of the contact using multi-frequency. Each material was placed in the same general location, at the center of the sensor.

In these experiments, the sensor was placed on a table. A 3D-printed object is placed on the sensor at the center. The object is printed with a round shape to match the roundness of the sensor and eliminate factors from edges. One face of the object is left untouched, thus having a plastic side, and the opposite side is covered with aluminium foil, thus having a metallic side. The thickness of the object is around 2 cm, thus isolating the metallic part of the plastic part. During the experiment, the object is placed either on one side or the other to simulate a plastic or metallic touch. To simulate forces, we used precision weights. Each weight is placed on top of the object to simulate the corresponding force. The pressures are calculated using the surface in contact between the 3D-printed object and the sensor, and the measuring weight.

**Figure 5:**
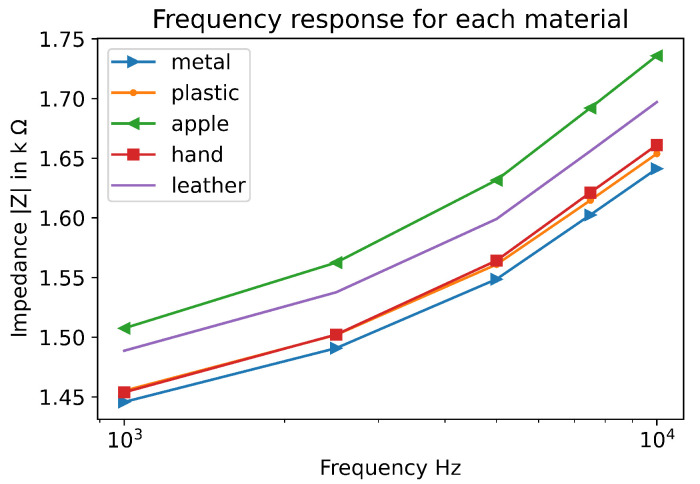
Impedance means for each contact type. The contact was made with the same pressure.

Figure 5 shows the response of each material at 10 kHz. The material was placed at the same place. The pressure applied was the same. The data represent the means of the impedance for each measurement. The results show material types can be deduced by looking at the raw data. However, the difference is very small for each material and other techniques can be used in order to discriminate more precisely the material type. For instance, delta between plastic and metal is only 16 Ω at 5 kHz. The learning method was used with these data and shows very good categorisation.

**Figure 6:**
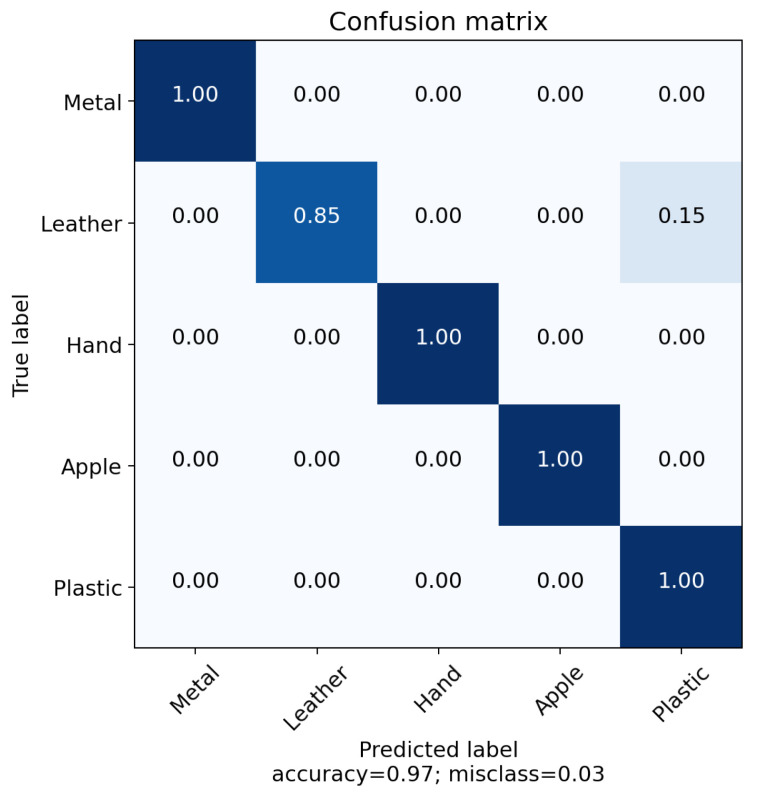
Confusion matrix of material recognition with constant temperature and pressure.

Figure 6 also shows the results of this experiment. Several materials were placed at the center. Each material was recognised with very good accuracy from different types. This shows that with the same temperature and pressure, material type is very well categorised.

### 4.1. Experiment 1: Pressure

Because the sensor is composed of Velostat, the fabric has a piezoresistive property. This property allows the sensor to change its electrical resistance in relation to the pressure applied. This is the main phenomenon allowing the pressure modality in such sensors. However, the question arises: how precisely the pressure can be retrieved?

**Figure 7:**
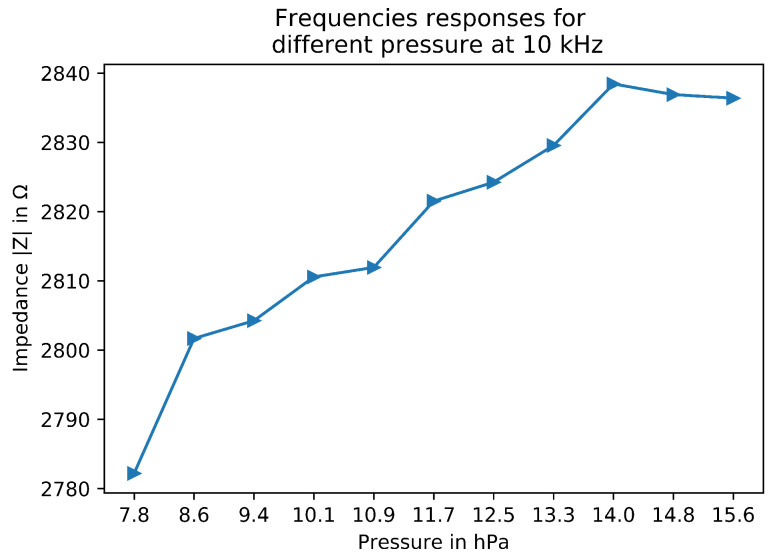
Impedance from different pressure with metal at 10 kHz.

In a second experiment, different pressures were applied to the center of the sensor, allowing averaging measurements for more robustness. Figure 7 presents the impedance mean for each measurement. It was conducted at 10 kHz. It shows an increase of impedance following higher pressures. This is mainly due to the piezoresistive effect of the fabric sensor. The more pressure applied, the more resistivity obtained from the sensor. Because of the constant current, the voltage needs to rise to compensate for the resistivity increase. By monitoring the tension measurement and its change with no pressure and some pressure, without any spatial reconstruction or other methods, the pressure can be retrieved. This allows gaining a modality by just looking at the raw data. However, this can be more difficult to retrieve if the contact is from a different material type, location or temperature. Nevertheless, this shows, with other works, that pressure is available from the electrical properties of the sensor itself.

The results also show two other things. First, there is a non-linearity between 7.8 hPa and 8.6 hPa measurements. This represents a weight of around 100 g. The hypothesis is that the sensor is not compressed enough concerning the Velostat and only “bends” it with the weight of the object. The second non-linearity is above 14.0 hPa. This starts a plateau in which the measurement does not increase over the pressure. This could be explained this time because the pressure reaches a threshold; the sensor cannot “bend” any more and the Velostat is fully compressed. The impedance reaches a maximum, like a Force-Sensing Resistor (FSR).

**Figure 8:**
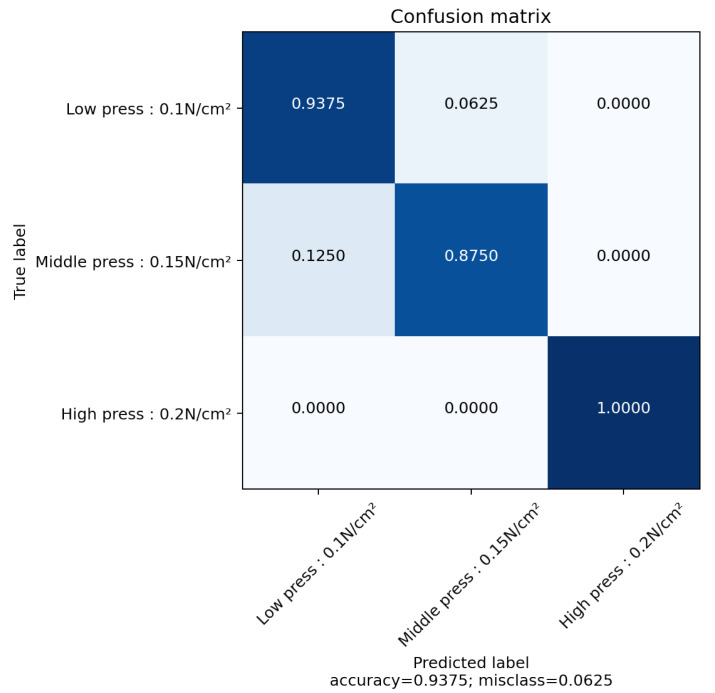
Confusion matrix of the PCA and k-means reconstruction with metal at different pressure.

Figure 8 shows the results of the pressure identification using metal contact at the same place with different levels of pressure: Low pressure between 0.05 N/cm${}^{2}$ (5 hPa) and 0.125 N/cm${}^{2}$ (12.5 hPa), middle pressure from 0.125 N/cm${}^{2}$ to 0.175 N/cm${}^{2}$ (17.5 hPa) and high pressure from 0.175 N/cm${}^{2}$ to 0.215 N/cm${}^{2}$ (21.5 hPa). The confusion matrix is filled by using the results of the PCA and k-means clustering against the labels. Pressure can be categorised without too many errors. More specifically, the errors only occur between the first two pressure categories. The accuracy is still above 90% between three levels of pressure. The pressure is obtained because of the material properties of the sensor. Even though the precise value of the pressure is not available, this can still be used to differentiate levels of pressures. This can be used to, for example, distinguish between harmful and safe pressures.

The applied pressure could be considered low for such a sensor but some limitations come from the architecture itself. The experiment needs to use precision weights to simulate force applied to the sensor. In order to accommodate all the weights, the object has to be large enough. This induces lower pressure. Nonetheless, the pressures are still representative for some use case, for early detection of touch in cobot for example. The Velostat itself possesses a pressure upper limit in which the impedance cannot change any more. One solution to tackle this issue is to change the sensing fabric. By making a piezoelectric fabric with specific properties, the pressure range can be changed.

### 4.2. Experiment 2: Temperature Influence

**Figure 9:**
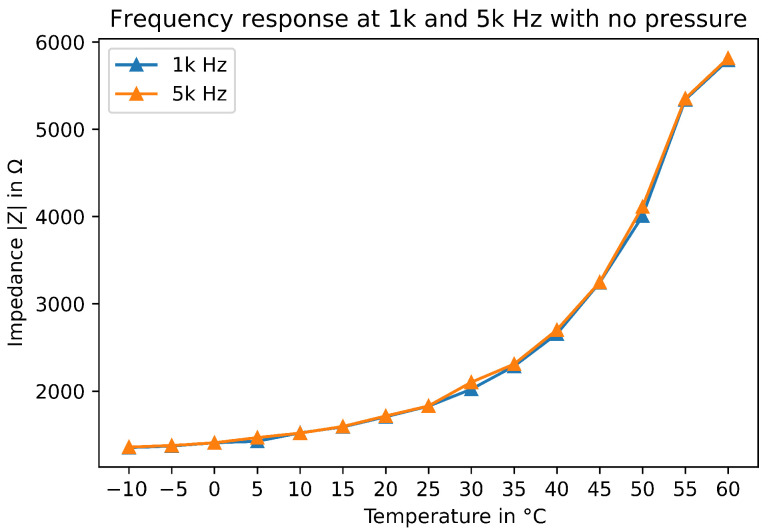
Impedance of the sensor for different temperatures with no contact.

In this experiment, the temperature was changed between acquisitions. The purpose is to describe the temperature influence of the measurements. Figure 9 shows the raw data of the EIT measurements with no contact at the sensor. The experiment was conducted in a controlled temperature chamber. The temperatures were chosen to be within the working range of the sensor fabric. The experiment starts at −10 °C up to 60 °C with 5 °C increment. As the figure shows, the tension follows an exponential growth. It grows almost the same for each frequency up until 75 kHz. This matches the fact that Velostat has a frequency range up to 100 kHz. Moreover, the exponential growth of the tension is stopped at 60 °C. After 65 °C, the Velostat starts changing its properties but can be reversed if the temperature goes down. However at 80 °C, the property changes are definitive. Our data show that impedance becomes greater due to increases in temperature. The fact that it grows so significantly is also a problem since it makes pressure reading difficult. A method of calibrating the sensor before measurement has to be carried out to obtain more reliable information. Therefore, by using a specific portion of the sensor, the calibration can be carried out to retrieve pressure with more accuracy. Moreover, thermal drift is a well know problem and compensation solution already exist in specific circuits (ASIC) components [43].

**Figure 10:**
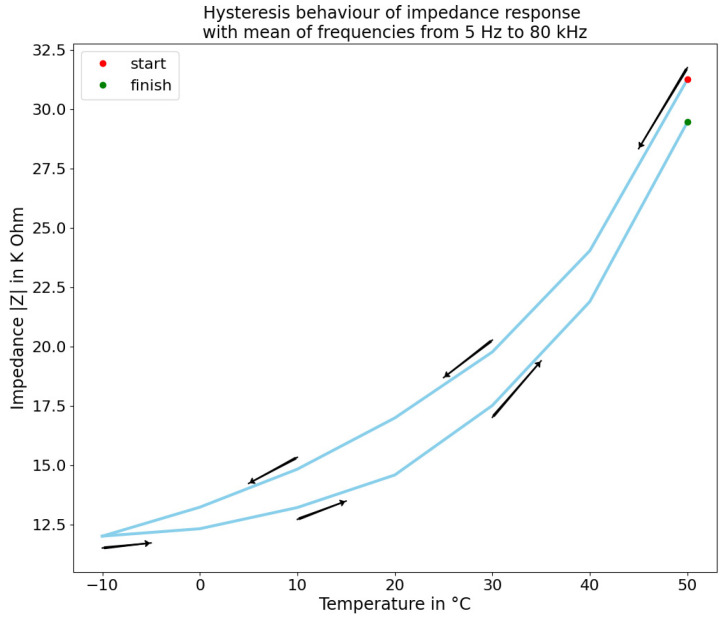
Hysteresis from impedance measurement of the sensor at different temperatures with frequency mean.

The experiment in Figure 10 shows the hysteresis of the fabric related to the temperature. All frequencies have been averaged for simplicity. The results show a strong hysteresis in the temperature range of the fabric. This behaviour is kept after several passes (or iterations) during the same time lapse. The time needed by the sensor to retrieve its original impedance value at rest (room temperature) surpasses the reasonable time between instances of contact. This for now cannot be changed and has to be compensated for. It also adds to the complexity associate of retrieving the pressure and a temperature change. Nonetheless, this shows how the temperature plays an important part in the impedance measurement. This can be used in order to either compensate temperature drift or retrieve temperature information.

### 4.3. Experiment 3: Material Type Recognition at Different Conditions

This experiment focused on the recognition of materiel type. The aim was to detect the material type using the sensor with our recognition model. The objects were placed at the same place. To minimise the effect of other factors, same pressures were applied to simulate similar weights. Temperature was constant through each experiment.

**Figure 11:**
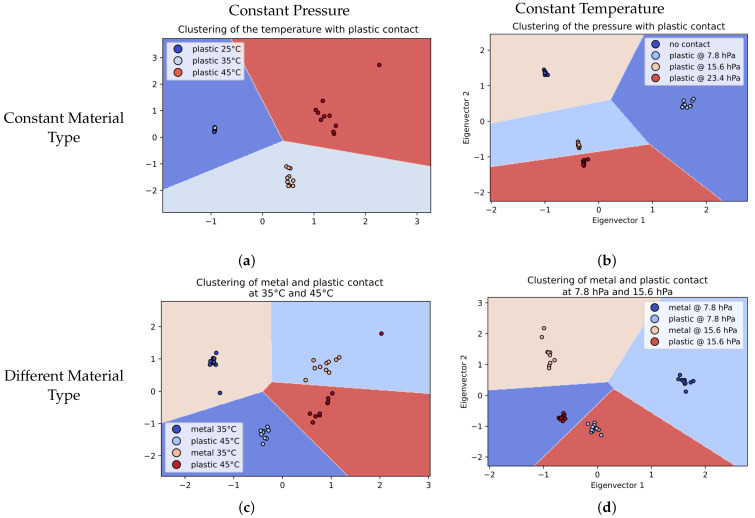
PCA transformed data with k-means clustering in different conditions, using all frequencies available for the PCA transformation: plot (**a**) the identification with plastic at different temperatures but with constant pressure (weight of 100 g producing 7.8 hPa); (**b**) the same plastic contact but at a constant temperature (25 °C) with different weights applied (100 g, 200 g and 300 g, producing up to 23.4 of hPa); (**c**) the identification of metal and plastic at two temperatures with constant pressure (7.8 hPa); and (**d**) the metal/plastic identification at two pressures with constant temperature (25 °C).

Figure 11 shows four different cases. Each one was used to differentiate only one parameter. For each figure, the color labels correspond to the dot plot, whereas the background color is only used to illustrate the decision boundaries of the k-means. The color label is the ground truth and is used to check the performances of the categorisation. Figure 11a shows how the model can differentiate the temperature with the same material type. This demonstrates once again how the temperature can be retrieved with our sensor. Figure 11b shows, on the other hand, how the sensor behaves with different pressures at a constant temperature with the same material type. In both cases, the pressure and temperature are retrieved with the same method and can be categorised well. Overall, the results show the model is robust and can be used to categorise different modalities. Other models based on supervised learning can be used to retrieve more preciselythe pressure and temperature values but by keeping the model exactly the same, different modalities can still be retrieved simultaneously. 

The next experiment was conducted using two samples of the same size: one of plastic and one of metal. Each sample was pressed with two pressure levels: 7.8 hPa and 23.4 hPa. Each acquisition was achieved with only one sample in contact with the sensor. Figure 11d shows the results. The two pressures were all correctly categorised using the same model as previously. The results also show that material discrimination is still available with pressure discrimination. The sensor is able to distinguish if the contact is a 100 g metal or a 100 g plastic, for example.

Figure 11c is the result of the experiment conducted with metal and plastic at two different temperatures. The purpose of this experiment was to check if the material recognition is still working at different temperatures. The object was placed as usual next to the first electrode couple. The clustering manages to categorise all different types of stimuli. However, some data are not correctly categorised. This is because temperature plays such an important role in the impedance measurement. Nonetheless, the accuracy is still above 90% recognition.

**Figure 12:**
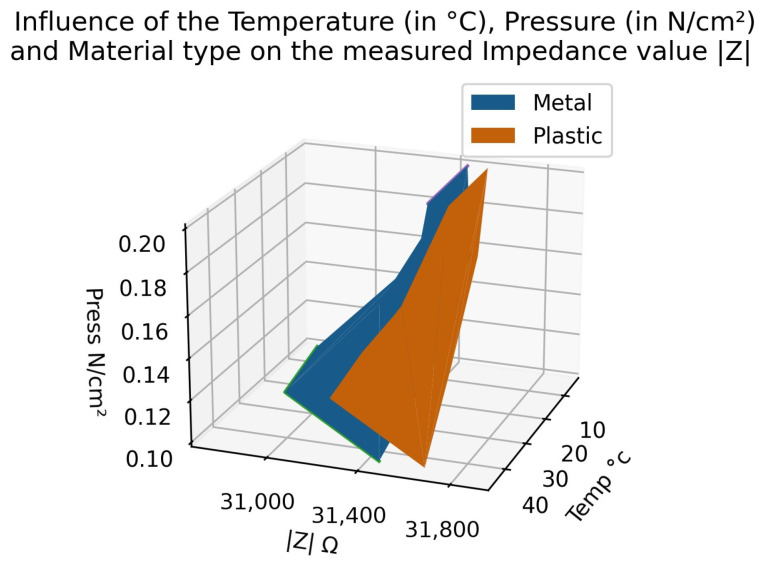
Summary of the influence of Temperature, Pressure and Material Type in the impedance value. Each plane corresponds to one particular material type (metal or plastic).

Figure 12 shows the different parameters influencing the measured impedance. The temperature, pressure and corresponding impedance are plotted in a 3D graph, each representing an axis. The measure is from an experiment using metal as a material type. Because the resultant plane is not straight in any direction, a classifier could distinguish between the two modalities. Moreover, by adding the plane from another material type, if the two planes did not cross each other, the material type could be distinguished as well. This figure shows how the device is capable of recognising material types, either the pressure or the temperature. This makes our sensor robust to environmental changes.

Furthermore, it also makes the sensor sensible enough to differentiate between the temperature and the pressure at the same time. The sensor can categorise at the same time the pressure, the temperature and the material type. The ranges of the modalities are from −20 °C up to 60 °C for the temperature, from 10 g up to 500 g (between 0.05 N/cm${}^{2}$ and 0.25 N/cm${}^{2}$) and differentiate between plastic and metal. This gives us the ability to develop a multi-modal sensor thanks to multi-frequency analysis.

## 5. Conclusions

This research presents a novel approach in developing multi-modal tactile sensors. By using a fabric with some electrical properties, new modalities can be retrieved. EIT is the base for the measurement but using new reconstruction algorithms, material type can be retrieved. This algorithm, based on machine learning, uses raw data and so can also be used to extend the scope of EIT. Pressure information can be retrieved directly from the raw data. By using some calibrations, the temperature hysteresis can be contained and pressure information/material type can be retrieved. In contrast, because of the great reaction of the temperature, the fabric itself can be used as a temperature sensor. Finally, a method can be developed in order to obtain all these modalities at once. This will provide a new way to develop human–machine interfaces such as in robotics. Our novel approach allowed us to use the same algorithm to obtain pressure as well as temperature while still being able to differentiate material types. Because spatial reconstruction is available, this sensor is capable using four modalities (pressure, temperature, material types and localisation). This implication allows one sensor to have all of this modalities, while being flexible and able to be scaled. With more than 95% accuracy in material recognition under different pressures and temperatures, the sensor shows its capabilities thanks to the contribution of the Multi-Frequency Analysis. The device is also compatible in real time. Even though it was not the focus of our research, experiments were conducted. Currently the online measurement is slow, taking up to 20 s per frame. Optimisation has been done by using simultaneous multiple frequencies down to 2 s per frame. We are currently designing and building a new acquisition card that will allow us to reduce even more the time for each frame to obtain an acquisition rate up to 50 Hz. The processing part is very fast because of the methods used, thus making the acquisition part the only challenge in terms of reaching a faster time. Moreover, the device is still affordable, with low energy consumption and low complexity. The challenge will be to obtain a robust measurement from this device. Having a good calibration unit is needed in order to compensate for the hysteresis. Other fabrics that share similar electrical properties with the ones used are also a viable solution. By finding a fabric with a small hysteresis, both in time and amplitude, the sensor will increase in accuracy. We use frequency per frequency implementation to obtain more precise raw data as well as to reduce the signal processing aspect. The next phase will be to implement simultaneous multi-frequency injection to speed up the process, thus allowing a full real-time functionality for future implementation.

## Figures and Tables

**Table 1:** Flexible tactile sensor systems and methods.

System	Material	Technique	Stimuli Injection	Modality	Frame Rate
Nagakubo et al., 2007 [24]	PSCR Multilayers Fabric	EIT	DC	Pressure/Localisation	1 kHz
Suen et al., 2018 [19]	PDMS/ZnO	3 × 3 grid	AC 1–10 Hz	Pressure/Temperature	NC
Lee et al., 2020 [25]	TENG/rGO	4 × 4 grid/KNN	NC	Pressure	NC
Di Giacomo et al., 2017 [26]	Pectin	4 × 4 grid	AC 5 Hz/DC	Temperature	50 Hz
Russo et al., 2017 [4]	Nylon coated with polypyrrole	EIT 16 electrodes	AC 2kHz/DC	localisation	78 Hz
Pang et al., 2020 [22]	Graphene Aerogel	10 × 10 grid/CNN	NC	Pressure/localisation	NC
Yoon et al., 2017 [27]	Carbon Elastomer	EIT 16 electrodes	DC	Pressure/Localisation	35 Hz
Oh et al., 2018 [28]	MWCNT-PDMS	EIT 24 electrodes	AC 1kHz	Pressure/Localisation	14 Hz
O’Neill et al., 2018 [29]	CNT-PDMS	4 electrods/RNN	NC	Pressure	30 Hz

## Data Availability

Not applicable.

## Abbreviations

The following abbreviations are used in this manuscript:

AC	Alternative Current
ASIC	Application Specific Integrated Circuit
DC	Direct Current
EIDORS	Electrical Impedance and Diffuse Optical Tomography Reconstruction Software
EIT	Electrical Impedance Tomography
EIS	Electrical Impedance Spectroscopy
ERT	Electrical Resistance Tomograpgy
GMM	Gaussian Mixture Model
ICU	Intensive Care Unit
MFA	Multi Frequency Analysisi
NC	Not Communicated
PCA	Principal Component Analysis
SVM	Support Vector Machine

## References

[1] Zou L., Ge C., Wang Z.J., Cretu E., Li X. (2017). Novel tactile sensor technology and smart tactile sensing systems: A review. Sensors.

[2] Sundaram S., Kellnhofer P., Li Y., Zhu J.Y., Torralba A., Matusik W. (2019). Learning the signatures of the human grasp using a scalable tactile glove. Nature.

[3] Kato Y., Mukai T., Hayakawa T., Shibata T. Tactile sensor without wire and sensing element in the tactile region based on EIT method. Proceedings of the IEEE Sensors.

[4] Russo S., Nefti-Meziani S., Carbonaro N., Tognetti A. (2017). Development of a high-speed current injection and voltage measurement system for electrical impedance tomography-based stretchable sensors. Technologies.

[5] Martinez-Cesteros J., Medrano-Sanchez C., Plaza-Garcia I., Igual-Catalan R., Albiol-Pérez S. (2021). A Velostat-Based Pressure-Sensitive Mat for Center-of-Pressure Measurements: A Preliminary Study. Int. J. Environ. Res. Public Health.

[6] Tawil D.S., Rye D., Velonaki M. (2011). Improved Image Reconstruction for an EIT-Based Sensitive Skin with Multiple Internal Electrodes. IEEE Trans. Robot..

[7] Hirata A., Takano Y., Kamimura Y., Fujiwara O. (2010). Effect of the averaging volume and algorithm on the in situ electric field for uniform electric-and magnetic-field exposures. Phys. Med. Biol..

[8] Lionheart W., Polydorides N., Borsic A. (2005). The reconstruction problem. Electr. Impedance Tomogr. Methods Hist. Appl..

[9] Abdelwahed M., Pitti A., Romain O., Ouezdou F.B. Use of Multi-frequency Electrical Impedance Tomography as Tactile Sensor for Material Discrimination. Proceedings of the 2020 5th International Conference on Advanced Robotics and Mechatronics (ICARM).

[10] Pugach G., Melnyk A., Tolochko O., Pitti A., Gaussier P. Touch-based admittance control of a robotic arm using neural learning of an artificial skin. Proceedings of the IEEE/RSJ International Conference on Intelligent Robots and Systems (IROS).

[11] Pugach G., Pitti A., Gaussier P. (2015). Neural learning of the topographic tactile sensory information of an artificial skin through a self-organizing map. Adv. Robot..

[12] Pugach G., Khomenko V., Melnyk A., Pitti A., Henaff P., Gaussier P. Electronic hardware design of a low cost tactile sensor device for physical Human-Robot Interactions. Proceedings of the IEEE XXXIII International Scientific Conference Electronics and Nanotechnology, ELNANO.

[13] Li X., Zhou Y., Wang J., Wang Q., Lu Y., Duan X., Sun Y., Zhang J., Liu Z. (2019). A novel deep neural network method for electrical impedance tomography. Trans. Inst. Meas. Control.

[14] Wu Y., Chen B., Liu K., Zhu C., Pan H., Jia J., Wu H., Yao J. (2021). Shape reconstruction with multiphase conductivity for electrical impedance tomography using improved convolutional neural network method. IEEE Sens. J..

[15] Ryun S., Kim J.S., Lee H., Chung C.K. (2017). Tactile frequency-specific high-gamma activities in human primary and secondary somatosensory cortices. Sci. Rep..

[16] Mountcastle V.B., Talbot W.H., Sakata H., Hyvärinen J. (1969). Cortical neuronal mechanisms in flutter-vibration studied in unanesthetized monkeys. Neuronal periodicity and frequency discrimination. J. Neurophysiol..

[17] Lamore P., Muijser H., Keemink C. (1986). Envelope detection of amplitude-modulated high-frequency sinusoidal signals by skin mechanoreceptors. J. Acoust. Soc. Am..

[18] Chambers M.R., Andres K., Duering M.V., Iggo A. (1972). The structure and function of the slowly adapting type II mechanoreceptor in hairy skin. Q. J. Exp. Physiol. Cogn. Med. Sci. Transl. Integr..

[19] Suen M.S., Lin Y.C., Chen R. (2018). A flexible multifunctional tactile sensor using interlocked zinc oxide nanorod arrays for artificial electronic skin. Sens. Actuators A Phys..

[20] Engel J., Chen N., Tucker C., Liu C., Kim S.H., Jones D. Flexible multimodal tactile sensing system for object identification. Proceedings of the SENSORS.

[21] Han S.T., Peng H., Sun Q., Venkatesh S., Chung K.S., Lau S.C., Zhou Y., Roy V. (2017). An overview of the development of flexible sensors. Adv. Mater..

[22] Pang K., Song X., Xu Z., Liu X., Liu Y., Zhong L., Peng Y., Wang J., Zhou J., Meng F. (2020). Hydroplastic foaming of graphene aerogels and artificially intelligent tactile sensors. Sci. Adv..

[23] Silvera-Tawil D., Rye D., Soleimani M., Velonaki M. (2015). Electrical Impedance Tomography for Artificial Sensitive Robotic Skin: A Review. IEEE Sens. J..

[24] Nagakubo A., Alirezaei H., Kuniyoshi Y. A deformable and deformation sensitive tactile distribution sensor. Proceedings of the IEEE International Conference on Robotics and Biomimetics (ROBIO).

[25] Lee Y., Ahn J.H. (2020). Biomimetic Tactile Sensors Based on Nanomaterials. ACS Nano.

[26] Di Giacomo R., Bonanomi L., Costanza V., Maresca B., Daraio C. (2017). Biomimetic temperature-sensing layer for artificial skins. Sci. Robot..

[27] Yoon S.H., Huo K., Zhang Y., Chen G., Paredes L., Chidambaram S., Ramani K. iSoft: A customizable soft sensor with real-time continuous contact and stretching sensing. Proceedings of the 30th Annual ACM Symposium on User Interface Software and Technology—UIST ’17.

[28] Oh J., Yang J.C., Kim J.O., Park H., Kwon S.Y., Lee S., Sim J.Y., Oh H.W., Kim J., Park S. (2018). Pressure Insensitive Strain Sensor with Facile Solution-Based Process for Tactile Sensing Applications. ACS Nano.

[29] Oâ€™Neill J., Lu J., Dockter R., Kowalewski T. (2018). Stretchable, Flexible, Scalable Smart Skin Sensors for Robotic Position and Force Estimation. Sensors.

[30] Voet A. (1981). Temperature effect of electrical resistivity of carbon black filled polymers. Rubber Chem. Technol..

[31] Yorkey T.J., Webster J.G., Tompkins W.J. (1987). Comparing reconstruction algorithms for electrical impedance tomography. IEEE Trans. Biomed. Eng..

[32] Barber D.C., Brown B.H. (1984). Applied potential tomography. J. Phys. Sci. Instrum..

[33] Barber D. (1989). A review of image reconstruction techniques for electrical impedance tomography. Med. Phys..

[34] Goren N., Avery J., Dowrick T., Mackle E., Witkowska-Wrobel A., Werring D., Holder D. (2018). Multi-frequency electrical impedance tomography and neuroimaging data in stroke patients. Sci. Data.

[35] Perrone A., Lapenna V., Piscitelli S. (2014). Electrical resistivity tomography technique for landslide investigation: A review. Earth-Sci. Rev..

[36] Adler A., Lionheart W.R.B. (2006). Uses and abuses of EIDORS: An extensible software base for EIT. Physiol. Meas..

[37] Adler A., Arnold J.H., Bayford R., Borsic A., Brown B., Dixon P., Faes T.J.C., Frerichs I., Gagnon H., GÃ¤rber Y. (2009). GREIT: A unified approach to 2D linear EIT reconstruction of lung images. Physiol. Meas..

[38] Bera T.K., Biswas S.K., Rajan K., Nagaraju J. (2011). Improving image quality in electrical impedance tomography (EIT) using projection error propagation-based regularization (PEPR) technique: A simulation study. J. Electr. Bioimpedance.

[39] Nissinen A., Heikkinen L., Kaipio J. (2007). The Bayesian approximation error approach for electrical impedance tomography—Experimental results. Meas. Sci. Technol..

[40] Dunlop M.M., Stuart A.M. (2015). The Bayesian formulation of EIT: Analysis and algorithms. arXiv.

[41] Hamilton S.J., Hauptmann A. (2018). Deep D-bar: Real-time electrical impedance tomography imaging with deep neural networks. IEEE Trans. Med. Imaging.

[42] Martin S., Choi C.T. (2015). Nonlinear electrical impedance tomography reconstruction using artificial neural networks and particle swarm optimization. IEEE Trans. Magn..

[43] Akbar M., Shanblatt M. (1992). Temperature compensation of piezoresistive pressure sensors. Sens. Actuators A Phys..

